# Albumin and interferon-β fusion protein serves as an effective vaccine adjuvant to enhance antigen-specific CD8+ T cell-mediated antitumor immunity

**DOI:** 10.1136/jitc-2021-004342

**Published:** 2022-04-22

**Authors:** Ssu-Hsueh Tseng, Max A Cheng, Emily Farmer, Louise Ferrall, Yu Jui Kung, Brandon Lam, Ling Lim, T-C Wu, Chien-Fu Hung

**Affiliations:** 1 Pathology, Johns Hopkins University, Baltimore, Maryland, USA; 2 Stanford Medicine, Stanford University School of Medicine, Stanford, California, USA; 3 Pathology, Oncology, Obstetrics and Gynecology, Molecular Microbiology and Immunology, Johns Hopkins University, Baltimore, Maryland, USA; 4 Pathology, Johns Hopkins Univ, Baltimore, Maryland, USA; 5 Oncology, Johns Hopkins University, Baltimore, MD, USA; 6 Obstetrics and Gynecology, Johns Hopkins University, Baltimore, MD, USA

**Keywords:** Immunotherapy, Adjuvants, Immunologic, CD8-Positive T-Lymphocytes

## Abstract

**Background:**

Type I interferons (IFN) promote dendritic cells maturation and subsequently enhance generation of antigen-specific CD8 +T cell for the control of tumor. Using type I interferons as an adjuvant to vaccination could prove to be a potent strategy. However, type I interferons have a short half-life. Albumin linked to a protein will prolong the half-life of the linked protein.

**Methods:**

In this study, we explored the fusion of albumin to IFNβ (Alb-IFNβ) for its functional activity both in vitro and in vivo. We determined the half-life of Alb-IFNβ following treatment in the serum, tumor, and tumor draining lymph nodes in both wild type and FcRn knockout mice. We characterized the ability of Alb-IFNβ to enhance antigen-specific CD8+ T cells using ovalbumin (OVA) or human papillomavirus (HPV) E7 long peptides. Next, we evaluated the therapeutic antitumor effect of coadministration of AlbIFNβ with antigenic peptides against HPVE7 expressing tumor and the treatment’s ability to generate HPVE7 antigen specific CD8+ T cells. The contribution of the antitumor effect by lymphocytes was also examined by an antibody depletion experiment. The ability of Alb-IFNβ to serve as an adjuvant was tested using clinical grade therapeutic protein-based HPV vaccine, TACIN.

**Results:**

Alb-IFNβ retains biological function and does not alter the biological activity of IFNβ. In addition, Alb-IFNβ extends half-life of IFNβ in serum, lymph nodes and tumor. The coadministration of Alb-IFNβ with OVA or HPVE7 antigenic peptides enhances antigen-specific CD8 +T cell immunity, and in a TC-1 tumor model results in a significant therapeutic antitumor effect. We found that CD8 +T cells and dendritic cells, but not CD4 +T cells, are important for the observed antitumor therapeutic effect mediated by Alb-IFNβ. Finally, Alb-IFNβ served as a potent adjuvant for TA-CIN for the treatment of HPV antigen expressing tumors.

**Conclusions:**

Overall, Alb-IFNβ serves as a potent adjuvant for enhancement of strong antigen-specific CD8 +T cell antitumor immunity, reduction of tumor burden, and increase in overall survival. Alb-IFNβ potentially can serve as an innovative adjuvant for the development of vaccines for the control of infectious disease and cancer.

**Key messages**

**What is already known on this topic**

- Interferon-β (IFNβ) is a major class of immune cytokine that can contribute to the efficacy of anticancer therapies; however the short half-life and inability to target key immune system tissues make IFNβ less clinically practical. Albumin (alb) is a ubiquitous plasma protein with a known extended half-life in vivo.

**What this study adds**

- This study describes a novel chimeric protein comprised of IFNβ fused to alb (Alb-IFNβ). The data presented here shows that fusing albumin to IFNβ can enhance the anti-tumor immune response, extend the half-life of IFNβ in vivo, and route it to lymph nodes and tumor.

**How this study may affect research, practice, or policy**

- This study describes a novel molecule Alb-IFNβ, which pending further research may be a prime candidate for clinical translation to deliver as an adjuvant therapy to patients with cancer.

## Introduction

Type I interferons are a major class of immune cytokines that also can be used as potent antiviral agents for the treatment of viruses such as hepatitis C.^1 2^ Beyond inducing antiviral immune responses, these cytokines, including both interferon-α (IFNα) and interferon-β (IFNβ), elicit a plethora of signals. For example, type I interferons contribute to the efficacy of anticancer therapies and have shown to mediate antineoplastic effects against many different malignancies.^3^ Type I interferons also intervene in different stages of cancer immunoediting, including the elimination of malignant cells via the immune system, the establishment of an equilibrium between the immune system and malignant cells, as well as during the phase in which neoplastic cell variants escape due to compromised immune systems.^3^ Most importantly, type I interferons have been shown to trigger dendritic cell (DC) maturation and migration toward lymph nodes (LNs), both of which are important in cross-priming cytotoxic immune responses.^3^ During antigen presentation by plasmacytoid DCs (pDCs), a high level of type I interferon secretion is observed in a concentrated area of T cells within lymphatic tissues associated with cancer. Recently, pDCs have been shown to traffic to tumor tissues and secrete chemokines such as C-X-C motif ligand 9 (CXCL9) and CXCL10, which recruit T cells to the tumor.^4^ Type I interferons can also directly increase the cytotoxicity and survival of CD8 +T cells.^5^

In recent years, immunotherapy has begun to emerge as a potentially promising strategy for cancer treatment. Additionally, many anticancer therapies that rely on type I interferon signaling have shown success in clinical use.^3 6^ In many instances, administering type I interferons can lead to antiviral and antiproliferative bioactivities.^7^ These cytokines even possess immunostimulatory functions.^7^ As an adjuvant to standard cancer immunotherapies, type I interferons have already demonstrated improvements in disease-free survival as well as overall survival. For example, several randomized trials using IFNα as an adjuvant in both low-dose and high-dose regimens have suggested effectiveness in improving survival outcomes of melanoma patients.^8–10^ Unfortunately, type I interferons have a short 2–3 hours long plasma half-life and require weekly injections when administered as an adjuvant,^10 11^ which significantly reduces its applicability in clinical settings.

Albumin is a ubiquitous plasma protein that is known for its long half-life in vivo and ability to thus increase the half-life of molecules that are associated with it.^12 13^ This is achieved via transcytotic recycling of albumin’s neonatal Fc receptor (FcRn).^14^ Due to its circulation pattern as a plasma protein physiologically, albumin is able to drain into the lymphatic tissues. Albumin binding has been shown to be effective for directing immunostimulatory molecules, including vaccine constructs, to the LNs in order to elicit potent immune responses.^15 16^ Albumin has low immunogenicity and is easy to construct, express, and purify, therefore it is an advantageous drug carrier.^17^ Albumin thus serves as a prime candidate to deliver cytokines and other biological cargo preferentially toward the LNs. Due to the ability of IFNβ to promote DC expansion^18^ and albumin’s ability to traffic toward LNs and extend half-life, we reason that a fusion between albumin and IFNβ may have a profound impact on cross-priming cytotoxic immune responses and may generate large pDC populations for antigen presentation. Strategies that expand cross-presenting DC populations also have the potential to be efficacious in the treatment of cancer.

We generated fusion protein albumin-IFNβ (Alb-IFNβ) by genetically fusing albumin to IFNβ. In this study, we evaluated the therapeutic potential of Alb-IFNβ to modulate immune cell phenotypes and improve antitumor responses. We show that Alb-IFNβ does not alter or impede the biological activity of IFNβ, as our novel molecule is able to generate DCs in vitro from bone marrow (BM) cells. The half-life of Alb-IFNβ is indeed longer than that of IFNβ alone, suggesting efficacy in the fusion strategy to albumin. In addition, in vivo distribution studies of Alb-IFNβ show preferential accumulation of our fusion protein in the tumor-draining LNs (tdLNs). More importantly, cross-presenting DCs generated by Alb-IFNβ in vivo are functional and able to generate potent antigen-specific T and B cell responses to both the ovalbumin (OVA) and human papillomavirus (HPV) E7 antigens. We also found that knocking out basic leucine zipper ATF-like transcription factor 3 (*Batf3*), which plays a crucial role in the development, expansion, and function of cross-presenting pDCs,^19 20^ reduced the antitumor effects of Alb-IFNβ. Furthermore, administrating Alb-IFNβ as an adjuvant to a clinical grade therapeutic HPV protein-based vaccine, TA-CIN, for the treatment of HPV-associated TC-1 tumors resulted in a significant reduction in TC-1 tumor burden, improved overall survival, and upregulation of E7-specific CD8 +T cell and DC activities. We also show that the antitumor immunity elicited by our fusion protein is both CD8- and DC-dependent and CD4-independent. In response to Alb-IFNβ, we observed an upregulation in CXCL9 and CXCL10 expressions in the tdLNs, which are chemoattractants secreted by DCs to recruit T cells to the tumor. Our results strongly support that Alb-IFNβ makes an excellent adjuvant to immunotherapies with strong therapeutic and clinical translation implications because it is able to enhance immunological responses mediated by DCs.

## Materials and methods

### Cells

As previously described, TC-1 cells express the HPV16 E6 and E7 proteins.^21^ Cells were grown in RPMI 1640 media, supplemented with 10% (v/v) fetal bovine serum, 50 units/mL of penicillin/streptomycin, 2 mM L-glutamine, 1 mM sodium pyruvate, 2 mM non-essential amino acids, and 0.1% (v/v) 2-mercaptoethanol at 37°C with 5% CO_2_. For the BMDC isolation and culture, the tibias and femurs were removed from C57BL/6 mice under sterile condition. Both ends of the bone were cut-off and BM was flushed out by 26-gage syringes with complete RPMI medium. Following red blood cell lysis and washing, BM cells were suspended with complete RPMI medium and seeded in 6-well culture plates with 29 ng/mL of granulocyte-macrophage colony-stimulating factor (GM-CSF) for 5 days.^22^ For the DC activation experiments, BMDCs matured in GM-CSF were treated with 0.1 µM of Alb-IFNβ, 0.1 µM of IFNβ, or 1 µg/mL of lipopolysaccharide (LPS) (as positive control) for 16 hours.

### Generation of Alb-IFNβ protein constructs

For the generation of pcDNA3- Alb-IFNβ, mouse IFNβ was first amplified via PCR with a cDNA template of the mouse IFNβ (pUNO1-mIFNB1) plasmid from Invivogen (San Diego, CA 92121 USA) and the following primers: 5′AAAGAATTCATCAACTATAAGCAGCTC-3’ and 5- AAACTTAAGTCAGTTTTGGAAGTTTCT-3’. The amplified product was then cloned into the EcoRI/Afl II sites of pcDNA3-Alb.^23^ The plasmid constructs were confirmed by DNA sequencing. Alb-IFNβ proteins were expressed using Expi293F expression system kit (Thermo Fisher Scientific, Waltham, Massachusetts, USA) according to manufacturer’s instructions. Expi293F cells were transfected with Alb-IFNβ. Proteins were purified by HiTrap Albumin column (GE Healthcare Life Sciences, Marlborough, Massachusetts, USA).

### Mice

Female C57BL/6 mice aged 6–8-weeks were purchased from Charles Rivers Laboratories (Frederick, Maryland, USA). All mice were maintained under specific pathogen-free conditions at the Johns Hopkins University School of Medicine Animal Facility (Baltimore, Maryland, USA). All animal procedures were performed according to protocols approved by the Johns Hopkins Institutional Animal Care and Use Committee. Recommendations for the proper use and care of laboratory animals were closely followed. For tumor inoculation, 2×10^5^ TC-1 cells in 50 µL of PBS were subcutaneously (s.c.) injected into 6–8 weeks old female C57BL/6 mice. Tumor volume was measured by digital calipers and greatest length and width were determined. Tumor volumes were calculated by the formula: tumor volume = (length × width^2^)/2. For CD4^+^ and CD8^+^ T cell depletion, 100 µg of anti-mouse CD8^+^ depleting antibody or 200 µg of anti-mouse CD4^+^ depleting antibody were administered to tumor-bearing mice for 3 days via intraperitoneal injection. The depletion was maintained through the experiment by giving depleting antibody once a week. CD4^+^ or CD8^+^ T cell depletion level were evaluated by flow cytometry on blood.^24^ Batf3-/- and FcRN-/- mice were acquired from Jackson Laboratories.

### Mice vaccination

Naïve or tumor-bearing C57BL/6 mice were s.c. vaccinated in the left rear flank twice at 1-week intervals with either 100 µg of OVA protein,^25 26^ 10 µg of HPV16 E7 long peptide (amino acids 43–62),^27 28^ or 25 µg of HPV vaccine TA-CIN^29^ in combination with or without either IFNβ (Prospecbio, East Brunswick NJ) or Alb-IFNβ twice at 1-week interval treatments. Vaccine doses were determined by previous experiments.^25–29^ One week after the final vaccination, peripheral blood mononuclear cells (PBMCs) were collected for flow cytometric analysis.

### Flow cytometry analyses

Peripheral blood samples from naïve and TC-1 tumor-bearing mice were collected into 100 µL of PBS containing 0.5 mM EDTA. Following red blood cell lysis and washing, PBMCs were collected and stained with Zombie Aqua to determine the cell viability. Fc receptors were blocked by anti-mouse CD16/CD32 antibody. To analyze OVA- and E7-specific T cells, Fc receptor blocked PBMCs were stain with PE-conjugated SIINFEKL (OVA) peptide or HPV16 E7aa49-57 peptide loaded H-2D^b^ E7 tetramer and FITC-conjugated anti-mouse CD8α antibody (Biolegend). To determine immune response of vaccinated tumor-bearing mice, TC-1 tumor-bearing mice were injected with either IFNβ or Alb-IFNβ followed by E7 long peptide as an adjuvant. One week after last vaccination, PBMCs were subsequently collected and stained for FITC-conjugated anti-mouse Ki67 antibodies, PE-conjugated HPV16 E7aa49-57 peptide-loaded H-2Db E7 tetramer, APC/Fire 750-conjugated anti-mouse CD8α antibodies, APC/Fire 750-conjugated anti-mouse CD11c antibodies, APC/Fire 750-conjugated anti-mouse I-A/I-E major histocompatibility II (MHC-II) antibodies, and PE-CF594 APC/Fire 750-conjugated anti-mouse CD86 antibodies for the presence of E7-specific CD8 +T cells and DCs. To evaluate biological function between Alb-IFNβ and IFNβ in vitro, FcRn-blocked BMDCs were stained with BV421-conjugated anti-mouse CD11c antibodies, APC/Fire 650-conjugated anti-mouse I-A/I-E (MHC II) antibodies, PerCP-conjugated anti-mouse CD40 and PE-CF594 -conjugated anti-mouse CD86. FACS analysis was performed using CytoFLEX S (Beckman) and data were analyzed by FlowJo software.

### Adaptive T cell transfer and tracking

C57BL/6 mice were s.c. injected with 5×10^5^ TC-1 cells for 10 days (after the tumor reached 8 to 10 mm in diameter). A 10 µg of IFNβ or 50 µg of Alb-IFNβ was intravenously injected into tumor-bearing mice through retro orbital sinus. One day after treatment with Alb- IFNβ or IFNβ, 5×10^6^ luciferase-expressing E7-specific T cells were intravenously injected into the tumor-bearing mice via tail vein. Luciferase-expressing E7-specific T cells were generated as previously described.^30^ For tracking the E7-specific T cell in tumor-bearing mice, 75 mg/kg of d-Luciferin was given to the mice via intraperitoneal injection and imaged by the IVIS Spectrum in vivo imaging system series 2000 (PerkinElmer) on day 1 and day 4 after T cell transfer. Total photon counts were quantified in the tumor site by using Living Image 2.50 software (PerkinElmer).

### ELISA

For the half-life experiment, naïve or TC-1 tumor-bearing C57BL/6 mice were intravenously injected with 10 µg of IFNβ or 50 µg of Alb-IFNβ. Serum were collected in EDTA-free Eppendorf tube on 3, 24 and 48 hours post-treatment. Tumor and LNs were harvested 16 hours post-treatment and then minced into 1–2 mm pieces and lysis by RIPA buffer (Cell Signaling Technology, Massachusetts, USA). IFNβ was measured by IFN beta Mouse ELISA Kit (Thermo Fisher Scientific) according to the manufacturer’s instructions. For the OVA and HPV16 L2 antibody detection, 1 ug/mL of mouse OVA or HPV16 L2 protein in PBS was coated on BRANDplates microplates overnight at 4°C. After 16 hours, the plates were washed, blocked with eBioscienceTM ELISA/ELISPOT Diluent (Thermo Fisher Scientific), and added diluted serum for 2 hours at room temperature.Non-vaccinated mice serum was used as control. Goat anti-mouse IgG-HRP secondary antibody was added at 1:5000 dilution for 1 hour, followed by TMB substrate. The OD at 450 nm was determined by 800 TS Absorbance Reader (BioTek Instruments).

### Quantitative real-time PCR

RNA was isolated from tumors treated with either PBS, IFNβ, or Alb-IFNβ, or from adjusted muscle tissues (as a normal tissue control) and reverse transcribed to cDNA. Gene expression levels were measured by quantitative real-time PCR (qRT-PCR) using SYBER Green with CXCL9-specific or CXCL10-specific primers. Briefly, total RNA was extracted by the Direct-zol RNA Kits (Zymo Research) following the manufacturer’s instructions. A 1 µL of RNA was converted to cDNA by the iScript Reverse Transcription Supermix (Bio-Rad Laboratories). A 1 µL of cDNA was used as template for qRT-PCR using SsoAdvanced Universal SYBR Green Supermix (Bio-Rad Laboratories), and qRT-PCR was performed using CFX96 Touch Real-Time PCR Detection System (Bio-Rad Laboratories). Primer for RT-PCR experiments to detect mouse CXCL9 expression, forward 5’- GAGCAGTGTGGAGTTCGAGG-3’; reverse 5’- TCCGGATCTAGGCAGGTTTG-3’, mouse CXCL10 forward 5’- GCCGTCATTTTCTGCCTCAT-3’; reverse 5’- GCTTCCCTATGGCCCTCATT-3’, 18S rRNA, forward 5’- GTAACCCGTTGAACCCCATT-3’; reverse 5’- CCATCCAATCGGTAGTAGCG-3’ (Integrated DNA Technologies). All data shown are normalized to the internal control gene 18S rRNA

### Statistical analysis

The statistical analysis was performed using GraphPad Prism V.6 software and data were interpreted as means with SD. Kaplan-Meier survival plots are used to estimate the survival percentage and tumor-free rate. Long rank tests were used to compare the survival time between treatment groups. Comparison between individual data points were used to analyze in the t- test and p value smaller than 0.05 is considered statistically significant, *p≤0.05, **p≤0.01,*** p≤0.001, **** p≤0.0001, ns=not significant.

## Results

### IFNβ fused to albumin retains biological function and does not alter the biological activity of IFNβ

**Figure 1:**
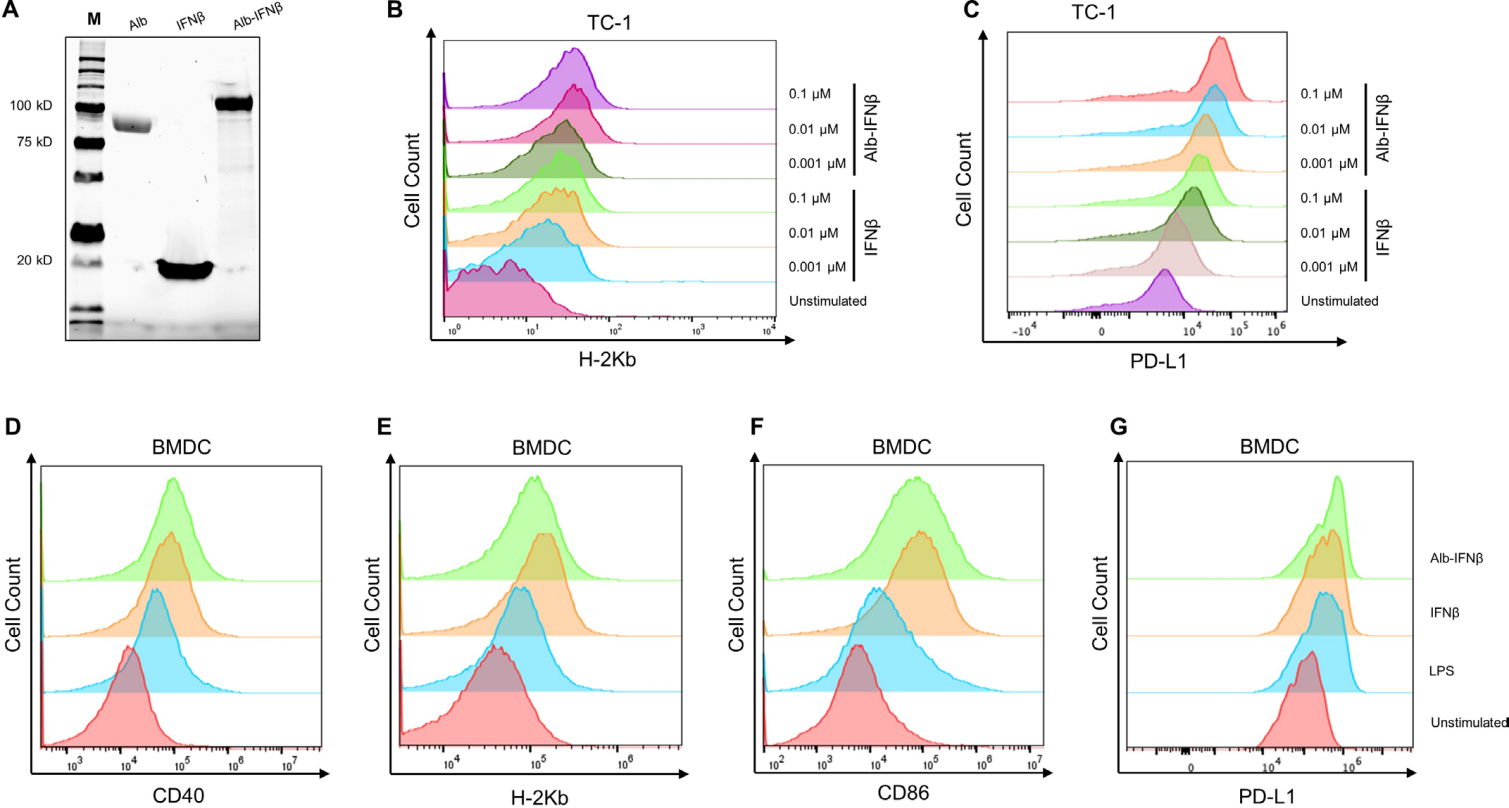
Characterization of biological activity of Alb-IFNβ compared with IFNβ using TC-1 cells or BMDCs. (A) SDS-PAGE analysis for purified albumin (Alb), IFNβ, and Alb-IFNβ. (M: molecular weight markers, as measured in kD). TC-1 cells were treated with 0.1 µM, 0.01 µM, or 0.001 µM of either IFNβ or Alb-IFNβ for 24 hours. The TC-1 cells were subsequently harvested and stained for (B) H-2Kb or (C) PD-L1 expression and analyzed by flow cytometry. Representative flow cytometry images of H-2Kb expression on TC-1 cells treated with either IFNβ or Alb-IFNβ. (D–G) BMDCs were treated with 0.1 µM of either IFNβ or Alb-IFNβ for 24 hours. BMDCs treated with lipopolysaccharide (LPS) as positive control. Shown here are representative flow cytometry images of (D) C40 expression, (E) H-2Kb expression, (F) CD86, and (G) PD-L1 expression on BMDCs treated with either IFNβ or Alb-IFNβ. BMDCs, bone marrow-dendritic cells. SDS-PAGE, sodium dodecyl sulfate–polyacrylamide gel electrophoresis

To bypass short half-life limitations posed by IFNβ, a genetic fusion protein consisting of albumin and IFNβ was produced and purified (figure 1A). In many instances, cytokine function can be altered when it is fused to a carrier protein. Thus, we determined whether the fusion of albumin to IFNβ (Alb-IFNβ) would affect the biological function of IFNβ. Through in vitro titration experiments, the expression of H-2Kb and PD-L1 on TC-1 cells increased as the cells were treated with increasing concentrations of IFNβ or Alb-IFNβ (figure 1B–C). BMDCs were also treated with either IFNβ alone or Alb-IFNβ, with BMDCs treated with LPS serving as a positive control and untreated BMDCs serving as a negative control. H-2Kb (figure 1E) and PD-L1 (figure 1G) expressions were comparable between Alb-IFNβ-treated BMDCs and BMDCs treated with IFNβ alone. Similarly, BMDCs treated with Alb-IFNβ also expressed similar levels of CD40 (figure 1D) and CD86 (figure 1F) compared with IFNβ alone. The addition of Alb-IFNβ failed to increase PD-1 or LAG3 expression using both TC-1 and BMDCs (online supplemental figure 1). Taken together, our data suggest that Alb-IFNβ retains similar biological function compared with IFNβ alone.

10.1136/jitc-2021-004342.supp1 Supplementary data

### The linkage of albumin to IFNβ extends half-life and increases IFNβ in serum, LNs and tumor in vivo

**Figure 2:**
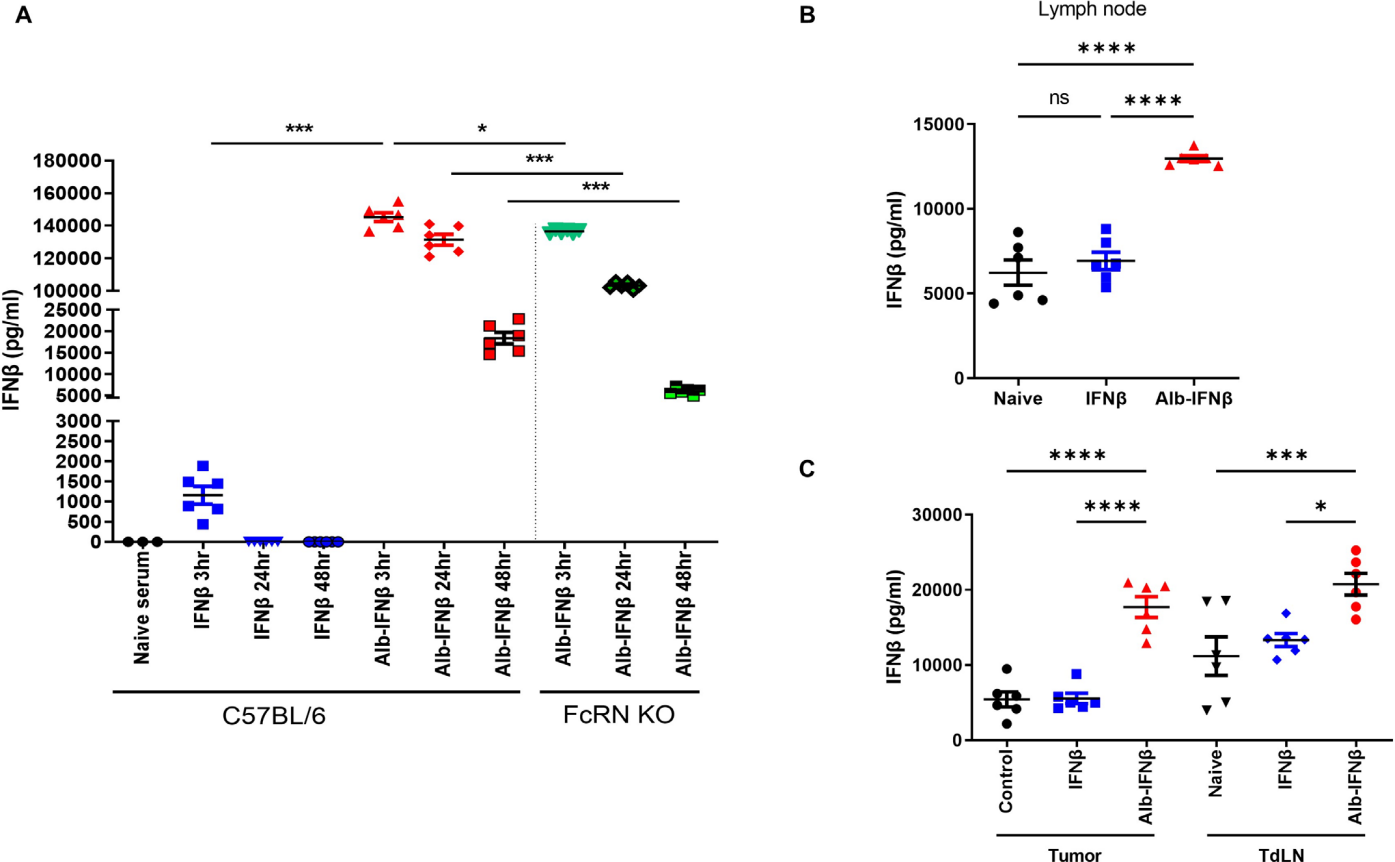
Analysis of Alb-IFNβ half-life within the C57BL/6 or FcRn knockout mice. (A) Naïve C57BL/6 or FcRN KO mice (6 mice per group) were intravenously injected with either IFNβ or Alb-IFNβ. Sera were collected 3, 24, and 48 hours post injection. An IFNβ ELISA assay was performed. (B) Naive C57BL/6 mice (6 mice per group) were injected with either IFNβ or Alb-IFNβ. LNs were collected 16 hours post injection. IFNβ concentration in LN protein extracts were detected by ELISA. Untreated mice LN lysates served as control. (C) TC-1 tumor-bearing mice (six per group) were treated with either IFNβ or Alb-IFNβ, tumors and tdLNs were harvested 16 hours post injection. IFNβ concentration in tumor lysates or LN lysate were detected by ELISA. Untreated mice tumor or LN lysate served as control. *P<0.05, ***p<0.001, ****p<0.0001. ns, not significant; tdLNs, tumor-draining lymph nodes.

Next, we sought to better understand the underlying trafficking mechanism of Alb-IFNβ in vivo. Albumin is known to increase the half-life of molecules that are associated with it through transcytotic recycling of the FcRn.^12–14^ Specifically, albumin fusion to IFNβ has been shown to increase the half-life of IFNβ by more thanfivefold.^31^ Thus, we suspected FcRn to play a role in our albumin fusion strategy. To determine the whether Alb-IFNβ has an extended half-life, we intravenously injected Alb-IFNβ or IFNβ into C57BL/6 mice. We found that levels of IFNβ were significantly higher at every time point when mice were treated with Alb-IFNβ as compared with mice treated with IFNβ alone (figure 2A). We also found that levels of IFNβ significantly decreased in FcRn knockout mice (figure 2A), suggesting the importance of FcRn in extending the half-life of Alb-IFNβ. Previous studies have also suggested that due to the circulation pattern of albumin in the plasma, albumin fusion proteins are able to preferentially traffic toward the draining LNs.^15 16^ From our experiments, we found that Alb-IFNβ was present at higher levels in LNs (figure 2B), and targets to both the tumors and the tumor draining LNs (tdLNs) more efficiently than IFNβ alone (figure 2C). Thus, we were able to confirm the notion that an albumin fusion strategy increases the half-life of conjugated IFNβ and is somewhat reliant on the presence of FcRn. We also showed that an albumin fusion strategy allows IFNβ to be targeted to both tumors and tdLNs at higher levels than being treated with IFNβ alone.

### Coadministration Alb-IFNβ and antigenic peptides enhances antigen-specific CD8+ T cell immunity in vivo

**Figure 3:**
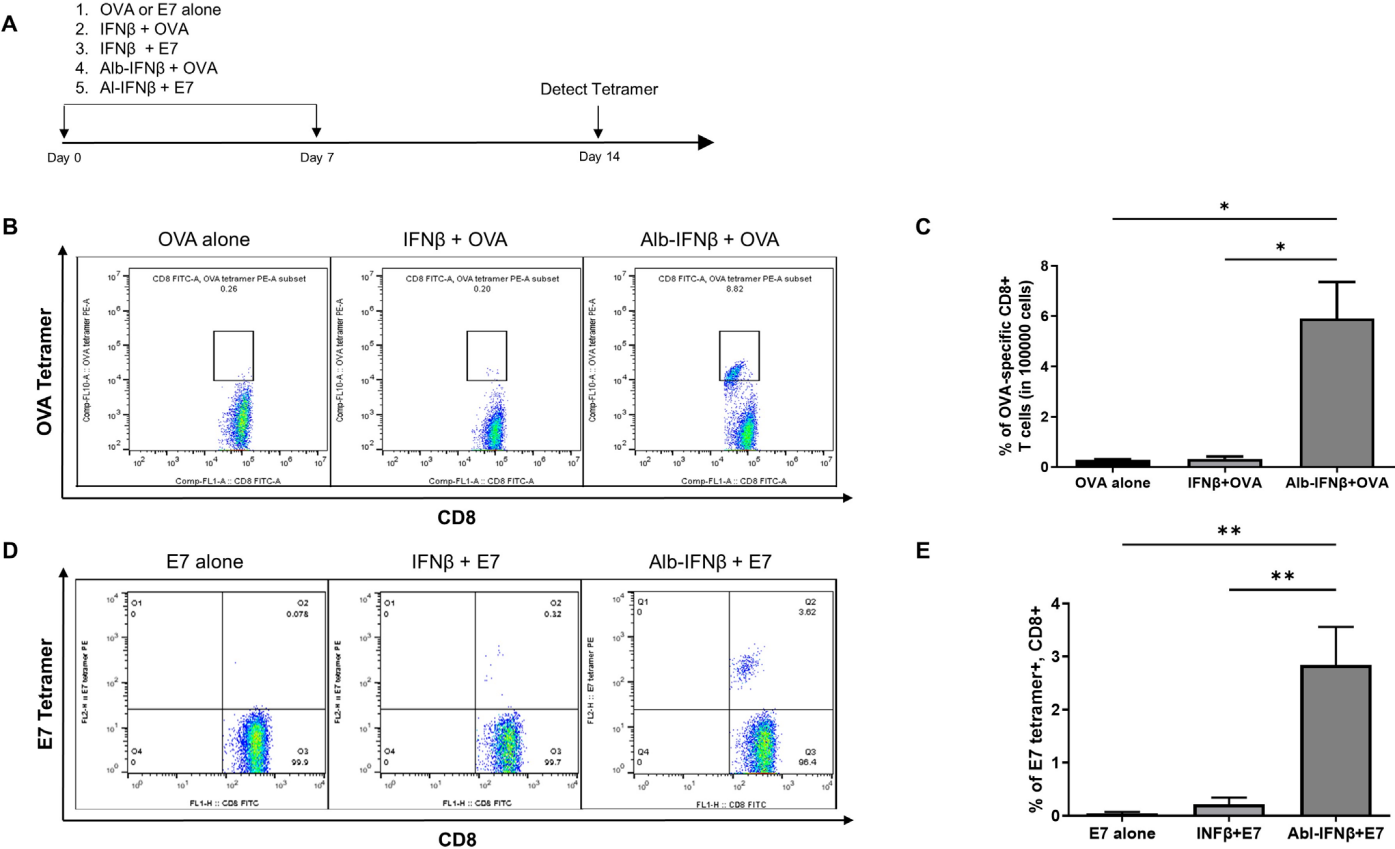
Characterization of the antigen specific CD8 +T cell immune response in mice treated with Alb-IFNβ mixed with OVA or E7. (A) Schematic illustration of the experiment. Briefly, naïve C57BL/6 mice (five per group) were subcutaneously vaccinated with antigen (either OVA protein or E7 long peptide (amino acids 43–62)) alone, or in combination with either IFNβ or Alb-IFNβ s.c. once a week at day 0 and 7. Seven days after the final vaccination, PBMCs were collected from previously-treated mice and stained with either PE-conjugated H-2 Kb OVA (SIINFEKL) or PE-conjugated HPV16 E7aa49-57 peptide-loaded H-2D^b^ E7 tetramer and FITC-conjugated anti-mouse CD8α antibodies, followed by flow cytometry analyses. (B) Representative flow cytometric images of OVA tetramer staining. (C) Bar graph summary of OVA tetramer staining. (D) Representative flow cytometric images of E7 tetramer staining. (E) Bar graph summary of E7 tetramer staining. *P<0.05, **p<0.01. OVA, ovalbumin; PBMCs, peripheral blood mononuclear cells; s.c, subcutaneously.

To evaluate the ability of Alb-IFNβ to promote CD8 +T cell responses to an exogenously-derived antigen, we vaccinated C57BL/6 mice with either OVA protein or E7 peptide (amino acids 43–62). It has been well documented that long E7 peptide is better than short peptides at inducing robust T cell responses, and it is capable of delivering specific cargo to antigen presenting cells.^32^ Additionally, OVA protein was used rather than OVA long peptide in order to demonstrate that our treatment strategy can be used with various vaccination platforms, such as protein vaccines. We simultaneously administered these antigens with or without either IFNβ or Alb-IFNβ to C57BL/6 mice twice at 1-week intervals (figure 3A). One week after the second vaccination, PBMCs were analyzed for the presence of OVA-specific and E7-specific CD8 +T cells by tetramer staining. Mice that received coadministration of Alb-IFNβ and OVA induced the highest number of OVA-specific CD8 +T cells compared with the other treatment groups (figure 3B–C). In addition, mice treated with coadministration of E7 long peptide and Alb-IFNβ similarly developed the most robust E7-specific CD8 +T cells (figure 3D–E). Finally, significantly higher titers of OVA-specific IgG2a/IgG1a antibodies were detected in the sera of mice vaccinated with both Alb-IFNβ and the OVA antigen compared with mice vaccinated with IFNβ and OVA or OVA alone (online supplemental figure 2). Our results suggest that coadministration of Alb-IFNβ with antigen enhances antigen-specific CD8 +T cell mediated and humoral immune responses in vivo compared with coadministration of IFNβ alone.

10.1136/jitc-2021-004342.supp2 Supplementary data

### Coadministration of Alb-IFNβ and E7 peptide generates a potent therapeutic antitumor effect against E7 expressing TC-1 tumor

**Figure 4:**
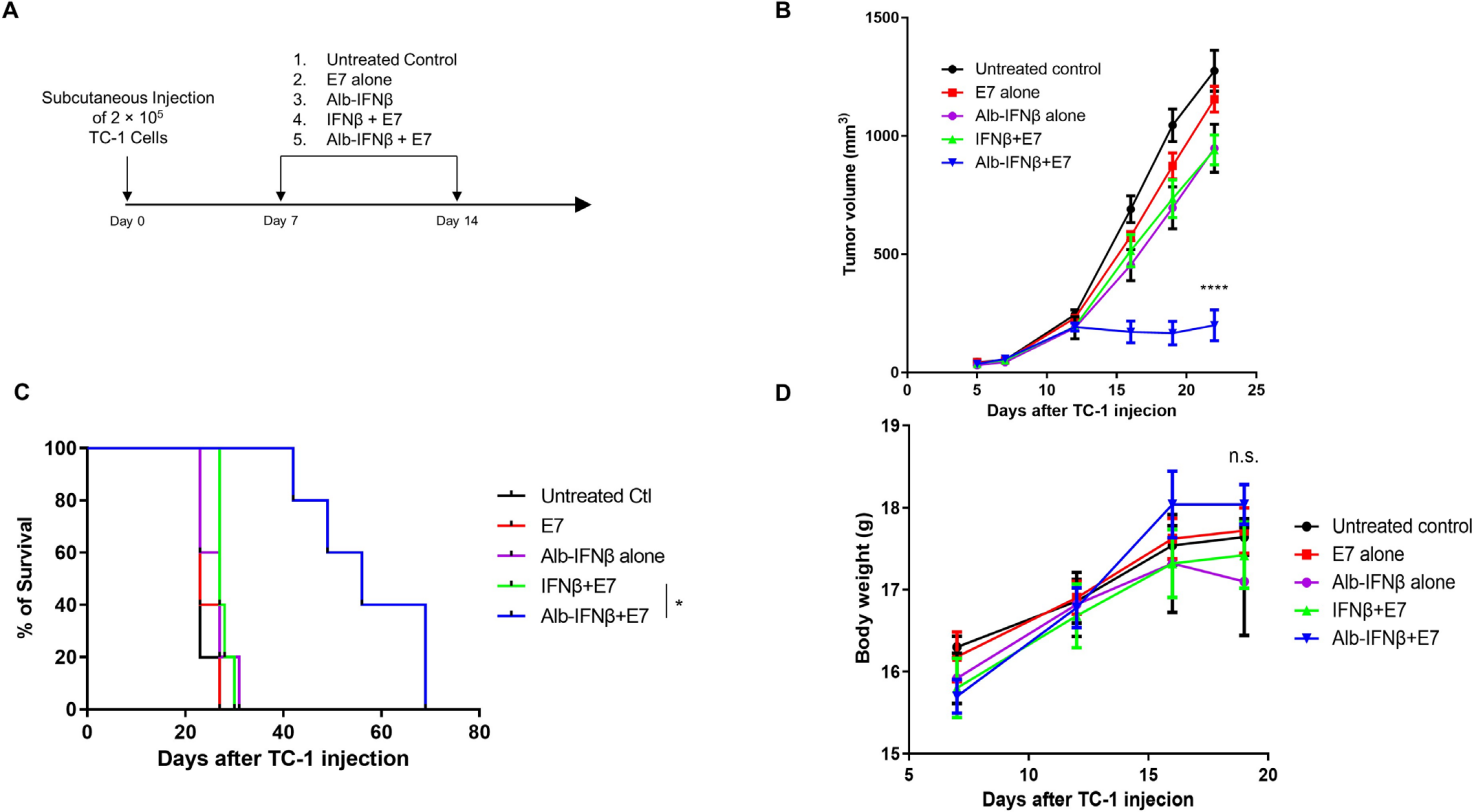
Characterization of therapeutic antitumor effect generated by Alb-IFNβ in conjunction with E7 vaccination. (A) Schematic illustration of the experiment. Briefly, C57BL/6 mice (five per group) were s.c. injected with 2×10^5^ TC-1 cells on day 0. These TC-1 tumor-bearing mice were then vaccinated with either E7 alone, Alb-IFNβ alone, IFNβ with E7, or Alb-IFNβ with E7 on days 7 and 14 after TC-1 tumor inoculation. A group of untreated TC-1 tumor-bearing mice served as the control group. (B) Tumor growth curve of TC-1 tumor-bearing mice. (C) Kaplan-Meier survival curve of TC-1 tumor-bearing mice. (D) Change in body weights of TC-1 tumor-bearing mice treated with the various therapeutic approaches. *P<0.05, ****p<0.0001. n.s, not significant; s.c, subcutaneously.

We next looked at the antitumor properties of Alb-IFNβ coadministered with E7 antigen. We treated C57BL/6 mice bearing HPV16 E7-positive TC-1 tumors with either E7 alone, Alb-IFNβ alone, E7 with IFNβ, or E7 with Alb-IFNβ twice at 1-week intervals (figure 4A). Tumor-bearing mice administered with the E7 antigen with Alb-IFNβ showed the smallest tumor volume compared with the other groups (figure 4B). Consistently, tumor-bearing mice administered with E7 with Alb-IFNβ survived twice as long compared with mice treated with the other treatment groups (figure 4C). Clinically, interferon has led to the development of many side effects and potentially toxic at high doses, especially when registered with multiple different treatments.^33 34^ While the doses of IFNβ and Alb-IFNβ used in this study were low and all tumor-bearing mice were only treated twice, there may still be toxicity concerns. One important side effect as a result of IFNβ-induced toxicity is weight loss.^35–38^ In our study, we did not observe any significant weight loss in tumor-bearing mice administered with any combination of the treatments (figure 4D). It seems that coadministration of Alb-IFNβ and E7 peptide does not induce any noticeable toxic side effects and was able to suppress tumor growth and prolong survival rates of tumor-beating mice better than tumor bearing mice treated with IFNβ and E7 peptide.

### Coadministration of Alb-IFNβ with HPV E7 peptides results in enhanced E7-specific CD8+ T cell immune responses and DC activity

**Figure 5:**
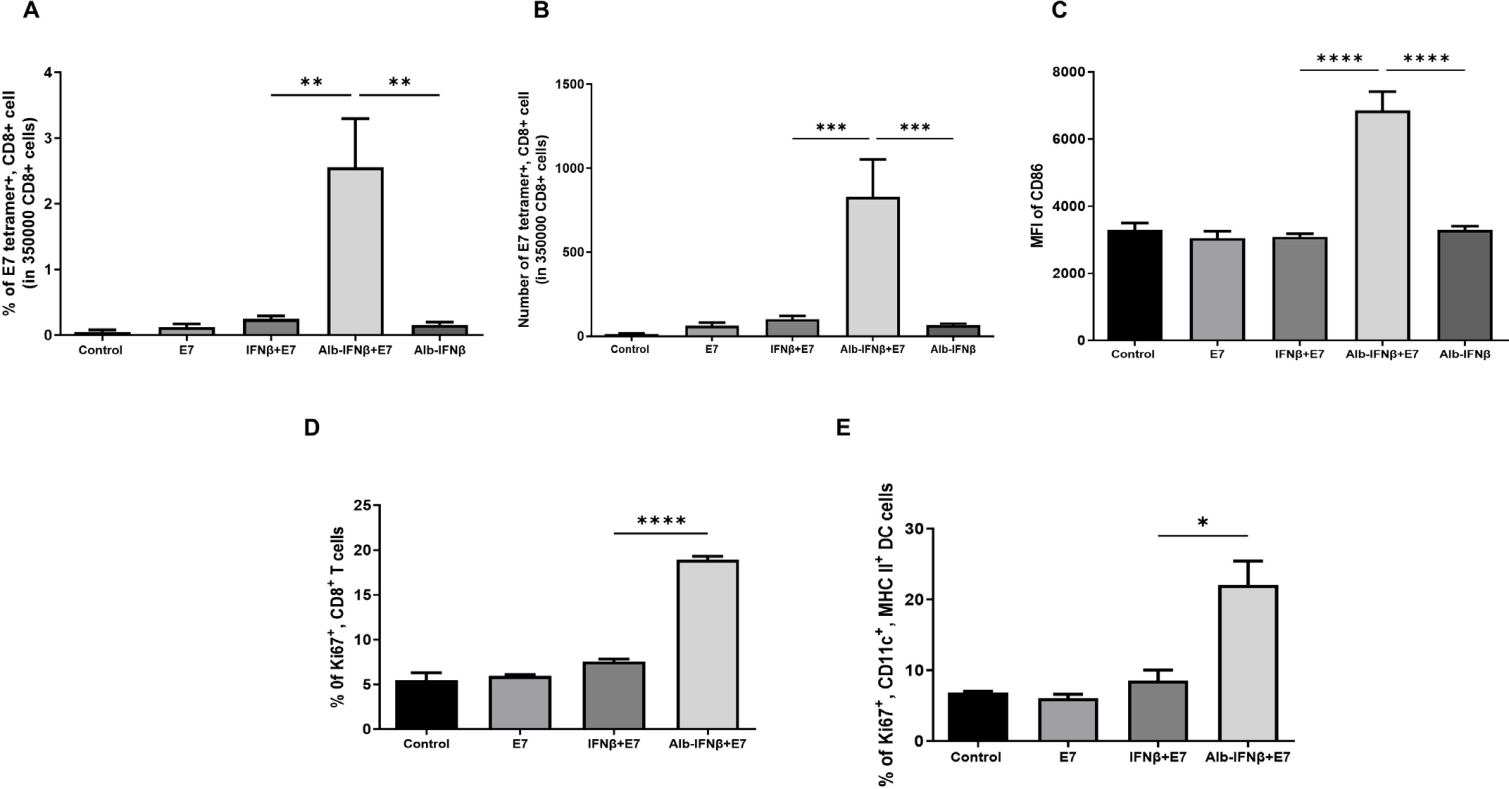
Characterization of E7-specific CD8 +T cells and DCs in PBMCs following treatment with Alb-IFNβ and E7 vaccination. (A–C) PBMCs were collected from the previously treated mice in figure 4 on 1 week after last vaccination (five per group), and stained with PE-conjugated HPV16 E7aa49-57 peptide-loaded H-2D^b^ E7 tetramer. (A) Bar graph summary of the percentages of E7 tetramer and CD8 +T cells in tumor-bearing mice administered with either E7, Alb-IFNβ, IFNβ with E7, or Alb-IFNβ with E7. (B) Bar graph summary of the total number of E7 tetramer and CD8 +T cells in tumor-bearing. (C) Bar graph summary of the mean fluorescence intensity of CD86 in CD11c+and I-A+/I-E +DCs. (D, E) PBMCs were collected from mice (five per group) treated with E7 alone, IFNβ with E7, or Alb-IFNβ with E7, which were independent from the mice used in A–C, and subsequently stained with FITC-conjugated anti-mouse Ki67 antibodies, APC/Fire 750-conjugated anti-mouse CD8α antibodies, APC/Fire 750-conjugated anti-mouse CD11c antibodies, and APC/Fire 750-conjugated anti-mouse I-A/I-E antibodies. (D) Bar graph summary of the percentages of proliferative Ki67 + and CD8+T cells. (E) Bar graph summary of percentage of proliferative Ki67+, CD11c+, and I-A+/I-E +DCs. *P<0.05, **p<0.01, ***p<0.001, ****p<0.0001. DC, dendritic cell; PBMCs, peripheral blood mononuclear cells.

Because albumin conjugation has been shown to extend the half-life of the IFNβ (figure 2), we explored whether a single s.c. administration of Alb-IFNβ with E7 peptide in tumor-bearing mice can elicit E7-specific CD8 +T cell-mediated immune responses and enhanced DC activity. DCs are known for their potent ability to cross present exogenous antigens to cytotoxic CD8 +T cells. While we have demonstrated that fusion of albumin to IFNβ does not impede the ability of IFNβ to expand DCs, we further examined whether coadministration of Alb-IFNβ with E7 antigen was superior in expanding DCs and promoting cytotoxic T cell responses to E7 antigens in vivo compared with coadministration of IFNβ with E7 antigen. TC-1 tumor bearing C57BL/6 mice were vaccinated with either E7 alone, Alb-IFNβ alone, IFNβ with E7, or Alb-IFNβ with E7. PBMCs were then collected from the mice for analysis. There was a significantly higher amount of E7-specific CD8 +T cells in mice treated with Alb-IFNβ and E7 compared with all other treatment conditions in the TC-1 tumor-bearing mice, indicating potent expansion of E7-specific CD8 +T cells following coadministration with Alb-IFNβ with E7 antigen (figure 5A–B). Moreover, mice treatment with Alb-IFNβ and E7 also had the highest levels of DC activation marker CD86 (figure 5C). In order to characterize the immune cell proliferation, we next examined the proliferative marker Ki67 following treatment.^39^ The advantage of using Ki67 for lymphocyte proliferative assays is to indicate the function of E7 specific T cells. Alb-IFNβ and E7 vaccinated mice also exhibited the highest proliferation activity of E7-specific CD8 +T cells in tumor-bearing mice compared with all other vaccination regimens (figure 5D). DCs in Alb-IFNβ and E7 vaccinated mice also had significantly higher Ki67 proliferative expression than other treatment conditions (figure 5E). Thus, our data suggest that Alb-IFNβ is able to generate and expand more potent antigen-specific cytotoxic T cell and DCs compared with IFNβ when coadministered tumor antigen in tumor-bearing mice.

### CD8 +T cells and cross presenting DCs, but not CD4 +T cells, are important for the observed antitumor therapeutic effect mediated by Alb-IFNβ coadministered with E7 peptides

**Figure 6:**
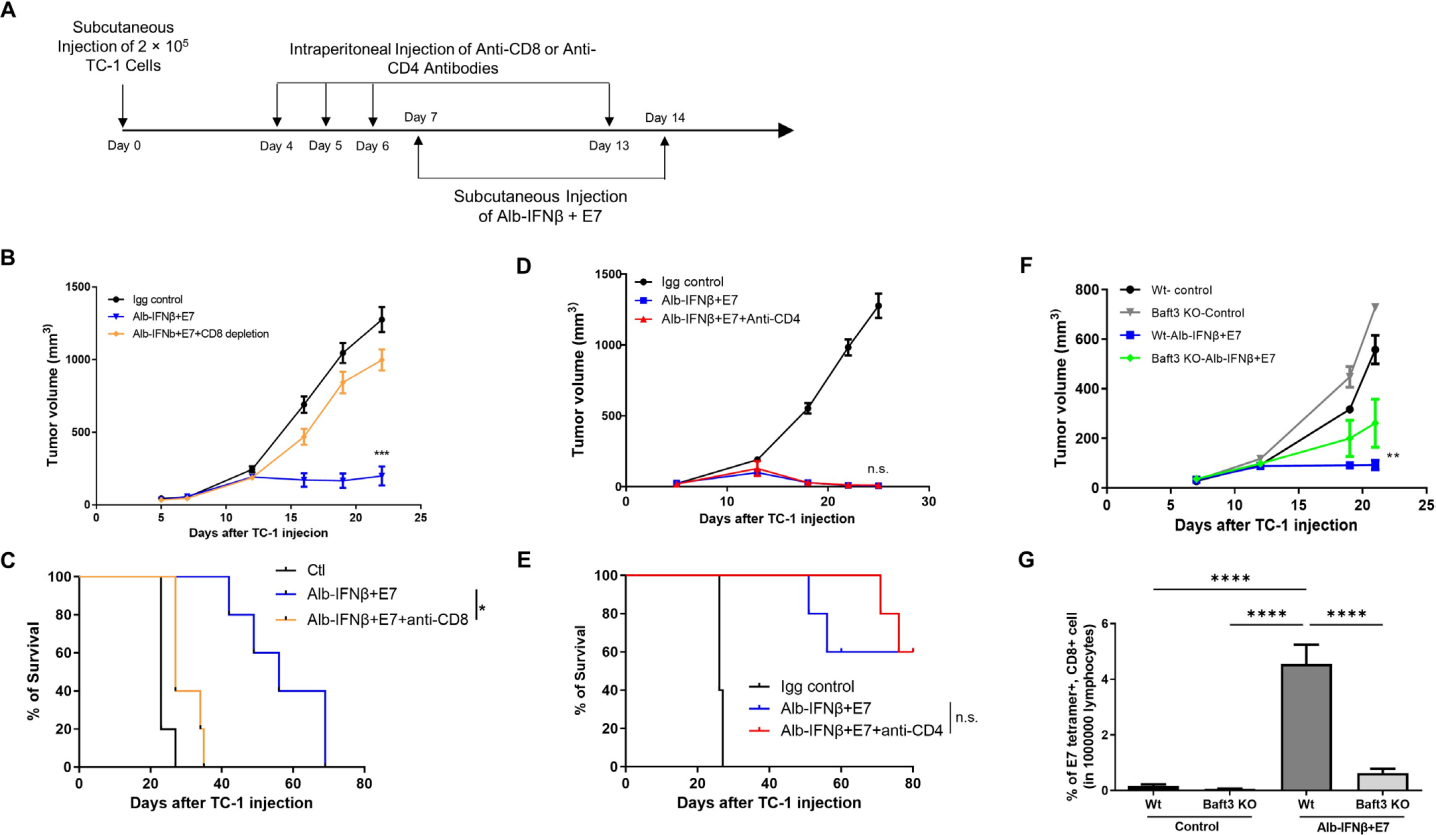
Determination of the role of CD8 T cells, CD4 T cells, or dendritic cells on therapeutic antitumor immunity generated by Alb-IFNβ and E7 vaccination. (A) Schematic illustration of the experiment. To deplete CD4 +or CD8+T cells in TC-1 tumor-bearing mice (five per group), C57BL/6 mice received either 200 µg of anti-mouse CD4 antibodies or 100 µg of anti-mouse CD8 antibodies daily by intraperitoneal injection for three continuous days prior to Alb-IFNβ treatment. Control mice received the same dose of mouse IgG isotype antibodies. (B) Tumor growth curve of CD8 +T cell-depleted mice. (C) Kaplan-Meier survival of CD8 +T cell-depleted mice. (D) Tumor growth curve of CD4 +T cell-depleted mice. (E) Kaplan-Meier survival of CD4 +T cell-depleted mice. To determine the significance of DC cells capable of cross presentation for the antitumor effect in TC-1 tumor bearing mice, Baft3 KO mice were used. (F) Tumor growth curve of Baft3 KO mice (G) Bar graph summary of the percentages of E7 tetramer and CD8 +T cells in Baft3 KO or control tumor-bearing mice administered with Alb-IFNβ with E7. *P<0.05, **p<0.01, ***p<0.001, ****p<0.0001. DC, dendritic cell; n.s, not significant.

After determining Alb-IFNβ elicits antitumor immunity through the expansion of both DCs and E7-specific CD8 +T cells when coadministered with E7 antigen, we wanted to test for the subset of lymphocytes that are essential for Alb-IFNβ to produce its effects. We first depleted either CD4 +or CD8+T cells in TC-1 tumor-bearing mice (n=5) by injecting them with either 200 µg of anti-mouse CD4 antibodies or 100 µg of anti-mouse CD8 antibodies daily for 3 days, followed by Alb-IFNβ and E7 vaccination (figure 6A). Our results suggest that CD8 +T cell depletion completely abolished the anti-tumor effects generated by Alb-IFNβ and E7 vaccination (figure 6B) and decreases the survival rate of Alb-IFNβ and E7 vaccinated tumor-bearing mice (figure 6C). However, the tumor volume and survival rates of Alb-IFNβ and E7 -treated tumor-bearing mice did not significantly change by depletion of CD4 +T cells (figure 6D–E). Furthermore, *Batf3* is known to play an important role in the development, expansion, and function of cross-presenting DCs.^15^ Thus, we perform the anti-tumor experiment in *Baft3* KO mice to determine whether it affected antitumor effect of Alb-IFNβ and E7 vaccination. *Batf3* KO mice treated with Alb-IFNβ and E7 vaccination apparently reduced their ability to control the tumor progression (figure 6F) and generated fewer E7-specific CD8 +T cells (figure 6G). Taken together, our results suggest both E7-specific CD8 +T cells and cross-presenting DCs are important for Alb-IFNβ to properly elicit potent antitumor responses when coadministered with E7 antigen.

### Treatment with Alb-IFNβ increased antigen-specific CD8+ T lymphocytes in the tumor microenvironment

To understand how Alb-IFNβ affects antigen-specific CD8 +T cells trafficking to the tumor microenvironment (TME), tumor-bearing mice were treated with either Alb-IFNβ, IFNβ, or PBS control followed by adaptive transfer of luciferase-expressing E7-specific CD8 +T cells (see online supplemental figure 3). By day 4, E7-specific CD8 +T cells were highly accumulated in the tumor area of mice administered with Alb-IFNβ compared with IFNβ (online supplemental figure 3). In comparison, tumor bearing mice administered with IFNβ did not demonstrated impact to the number of E7-specific CD8 +T cell in the tumor compared with untreated group. Taken together, our data indicated that administration of Alb-IFNβ facilitates tumor infiltration of E7-specific CD8 +T lymphocytes in the TME.

10.1136/jitc-2021-004342.supp3 Supplementary data

### Treatment with Alb-IFNβ leads to increased levels of chemokines in tumors and increased CD8+ T cell activity and DC activation in the tdLNs

Cross-presenting DCs have been shown to secrete chemokines such as CXCL9 and CXCL10. These chemokines are then able to recruit T cells to the TME, thus mounting an antitumor immune response.^4^ To test whether Alb-IFNβ can promote the expression of these chemokines in the tumors, we analyzed DC activation in the TME and changes in chemokine expression following Alb-IFNβ treatment. The levels of CXCL10 and CXCL9 were significantly higher in tumors treated with Alb-IFNβ compared mice treated with to IFNβ (online supplemental figure 4A-B). Within the tdLNs, mice treated with Alb-IFNβ exhibited significantly higher CD8 +T cell proliferative activity in the tdLNs of tumor-bearing mice compared with untreated control mice. However, there is no significant difference between mice treated with Alb-IFNβ or IFNβ (online supplemental figure 4C). Additionally, Alb-IFNβ was able to induce higher DC activation in the tdLNs compared with IFNβ (online supplemental figure 4D).Thus, our experiments showed that Alb-IFNβ treatment successfully increases chemokine expression, which also increases CD8 +T cell activity and DC maturation in the tdLNs of tumor bearing mice.

10.1136/jitc-2021-004342.supp4 Supplementary data

### Alb-IFNβ serves as a potent adjuvant for HPV protein based therapeutic vaccine, TA-CIN, for the treatment of HPV antigen expressing tumors

**Figure 7:**
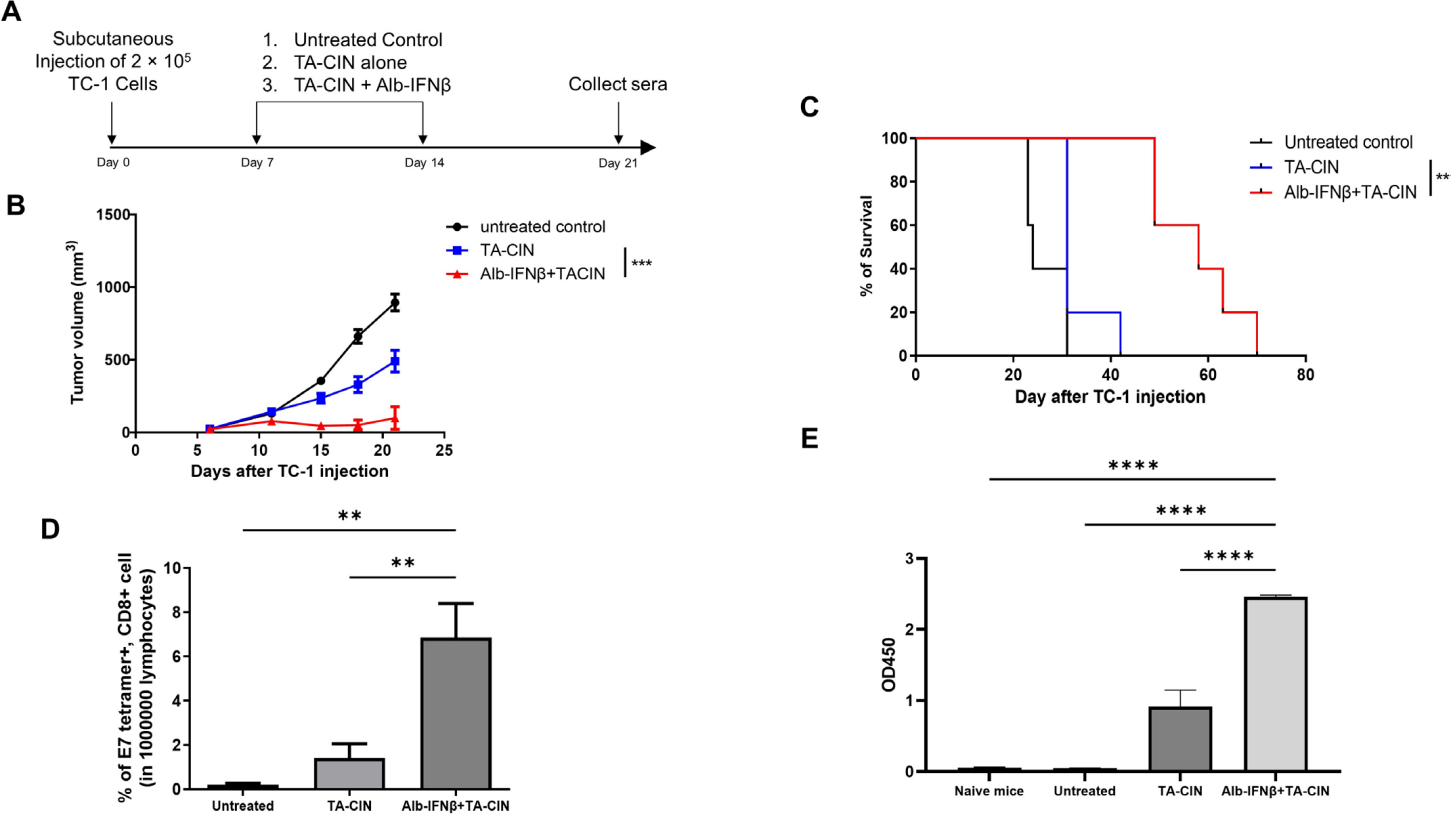
Characterization of therapeutic anti TC-1 tumor effect generated by Alb-IFNβ in combination with TA-CIN. (A) Schematic illustration of the experiment. C57BL/6 mice (five per group) were s.c. injected with 2×10^5^ TC-1 cells on day 0. 7 days after TC-1 injection, TA-CIN alone or in combination with Alb-IFNβ was s.c. injected into the mice every week for 2 weeks. Untreated TC-1 tumor-bearing mice served as control. (B) Tumor growth curve of TC-1 tumor-bearing mice. (C) Kaplan-Meier survival curve of TC-1 tumor-bearing mice. (D) Bar graph summary of the percentage of E7 tetramer and CD8 +T cells in tumor-bearing mice administered with either TA-CIN or Alb-IFNβ and TA-CIN. Untreated mice served as control. (E) Bar graph summary of the ELISA assay to detect mouse anti-L2 IgG2A antibodies in vaccinated mice. Sera from vaccinated and untreated mice were collected on day 21. Sera from naïve mice served as negative control. The sera were diluted by 1000-fold. **p<0.01, ***p<0.001, ****p<0.0001. s.c, subcutaneously. TA-CIN, Tissue Antigen-Cervical Intraepithelial Neoplasia

Tissue Antigen-Cervical Intraepithelial Neoplasia (TA-CIN) is a candidate therapeutic HPV protein vaccine comprised of a fusion of full length HPV16 L2, E6, and E7 proteins.^40^ It is administered as a filterable protein aggregate to promote uptake by antigen presenting cells. This protein vaccine has been shown to induce both E7-specific CD8 +T cell-mediated antitumor and HPV L2-specific neutralizing antibody responses in preclinical models.^16 40 41^ However, the clinical efficacy of TA-CIN alone may not be as effective as intended, probably due to the lack of immunogenic adjuvants in the formulation of the protein vaccine.^42 43^ Thus, we sought to overcome TA-CIN immunogenic deficiencies by combining it with Alb-IFNβ treatment. Tumor-bearing mice were administered either TA-CIN or TA-CIN in combination with Alb-IFNβ (figure 7A). TC-1 tumor-bearing mice receiving TA-CIN treatment in combination with Alb-IFNβ had significantly lower tumor growth compared with mice that were vaccinated with TA-CIN alone (figure 7B). Additionally, combination treatment of TA-CIN and Alb-IFNβ was also more effective in prolonging the survival of tumor-bearing mice than TA-CIN alone or untreated tumor-bearing mice (figure 7C). Combination treatment of Alb-IFNβ and TA-CIN also induced higher levels of E7-specific CD8 +T cells in tumor-bearing mice (figure 7D). Significantly higher level of anti-L2 IgG antibodies were similarly detected in the combination group compare to TA-CIN alone (figure 7E). Taken together, we show that Alb-IFNβ is able to enhance TA-CIN-elicited antitumor effects to suppress tumor growth and we believe that Alb-IFNβ serves as a potentially potent immunologic adjuvant.

## Discussion

In this study, we evaluated the therapeutic potential of Alb-IFNβ in combination with antigens to modulate immune cells and improve antigen-specific antitumor responses. Our data shown Alb-IFNβ not only retains similar biological activity compared with IFNβ in vitro but is able to generate potent antigen-specific T and B cell responses to OVA and HPV16 proteins. Additionally, vaccination of Alb-IFNβ and HPV16 antigens in tumor-bearing mice resulted in a significant reduction in tumor burden and better overall survival. The antitumor immune responses generated by Alb-IFNβ and HPV16 antigens vaccination were found to be CD8-dependent and DC-dependent and CD4-independent. One possible explanation for why CD4 +T cells are not as important for the antitumor effect is that it has been documented that mice vaccinated with IFNa and OVA antigens generate OVA specific CD8 independent of CD4 or CD40.^44^ This is likely because Alb-interferons cause maturation of DCs and also provide a third signal to enhance CD8 proliferation.^45^ We also observed a significant increase of the antigen-specific CD8 +T cells in the tumor location was observed in tumor bearing mice treated with Alb-IFNβ. Alb-IFNβ also accumulates in the tdLNs and facilitates the expansion of antigen-specific CD8 +CTLs in the TME. We suggested Alb-IFNβ can increase CD8 +T cell activities and promote DC maturation in the tdLNs possibly through inducing an upregulation of CXCL9 and CXCL10. An assessment of Alb-IFNβ used in combination with a clinical drug TA-CIN showed superior antitumor effects compared with TA-CIN alone, therefore suggesting Alb-IFNβ as an effective immunologic adjuvant. The therapeutic potential of Alb-IFNβ lead us to believe that it should be further investigated for clinical translation.

Alb-IFNβ holds immense therapeutic potential as a novel immunotherapy. With Alb-IFNβ we could possibly improve treatment schedules while limiting any side effects to generate potent antigen-specific antitumor responses. The linkage of Albumin to IFNβ not only extends half-life and but also leads to the targeting of IFNβ to the LNs and tumor in vivo and thereby serves as a potent adjuvant for vaccination. Thus, Alb-IFNβ can bypass shortcomings posed by the weekly administrations and increased dosages of IFNβ thereby limiting potential side effects in the clinic. Although PEGylated IFNα and IFNβ have also demonstrated increased half-lives in vivo, Alb- IFNβ could likely better target the interferons to LNs based on its natural circulation. Therefore, Alb-IFNβ has a higher chance of contacting immune cells in LNs, and subsequently enhanced the DC cross-presentation. However, future studies comparing the half-life and effectiveness of PEGylated IFNβ and Alb- IFNβ should be considered.

Anticancer immunotherapies harness the immune system to develop a response toward tumors. Immunotherapy can include checkpoint inhibitors, adoptive cell therapies, and cancer vaccines, among other approaches.^46 47^ There are many cancer vaccine delivery methods, including through intratumoral and localized mucosal routes.^48–51^ However, these delivery methods are invasive, therefore limiting the number of participants willing to take part in clinical settings.^52–54^ An alternative approach is to target tdLNs, which are known to accumulate tumor antigens that can be used to prime antitumor T cell responses.^55^ In our study, we show that Alb-IFNβ is able to target the tdLNs (figure 2). With our albumin-fusion targeting strategy, we can locally administer a less-invasive procedure that can target therapeutic vaccines toward the tdLNs in order to elicit potent, local antigen-specific antitumor responses within the TME (online supplemental figure S2). Alb-IFNβ therefore provides immense clinical opportunities to deliver antigens to DCs at the TME and expand robust cytotoxic immune responses. Additionally, in the current study we observed increased luciferase expressing antigen specific CD8 +T cells in the TME following treatment with Alb-IFNβ. There are at least two reasons that may account for the observed phenomenon. First, it may be attributed to the trafficking of the antigen specific CD8 +T cells to the location of the tumor (as implied by the study with CXCL9 and CXCL10). Second is that it may be due to the proliferation of antigen specific CD8 +T cells at the tumor location (as suggested by the characterization of Ki67).

In the current study, we have found that both *Batf3* is an important factor for the ability of Alb-IFNβ to control tumor progression. IFNβ enhances cross-presenting DC maturation, whereas *Batf3* is crucial to the development, expansion, and functioning of cross-presenting DCs.^15 56–58^ Thus, we used *Batf3* KO mice to study the role of this gene in the ability of Alb-IFNβ to expand cross-presenting DCs. We show that *Baft3* KO mice administered with Alb-IFNβ were less capable to control tumor growth progression and generated fewer E7-specific CD8 +T cells (figure 6). Of note, *Batf3* KO mice treated with Alb-IFNβ still were able to control tumor growth compared with untreated mice were, suggesting that although *Batf3* alone is important for Alb-IFNβ effect it is not the only contributing factor. Other factors may also contribute to the ability of Alb-IFNβ to control tumor in addition to *Batf3*.

In our study, we found that FcRn is an important mediator for the ability of albumin to extend the half-life of IFNβ. When administering Alb-IFNβ to FcRn KO mice, we noticed a shorter half-life of Alb-IFNβ compared with Alb-IFNβ in C57BL/6 mice (figure 2). However, despite a significant decrease in the half-life of Alb-IFNβ in FcRn KO mice, the half-life was still longer than the half-life of IFNβ in C57BL/6 mice. Thus, although FcRn can extend the half-life of IFNβ linked to albumin, some other factors may also contribute to the prolonged half-life mediated by albumin. It is of interest to further explore the other possible mechanisms that account for the prolongation of half-life of the protein fused to albumin.

We have observed tumor-bearing mice treated with Alb-IFNβ resulted in more tumor antigen specific CD8 +T cells in the tumor location (online supplemental figure S2). At least two reasons may account for the observed phenomenon. One is that the antigen specific T cells may be preferentially attracted to the tumor location in tumor-bearing mice treated with Alb-IFNβ. Alternatively, the other reason is that tumor-bearing mice treated with Alb-IFNβ results in enhanced proliferation of antigen specific CD8 +T cells in the tumor location. Indeed, our data from the characterization of CXCL9 and CXCL10 in the tumor location appears to be higher (online supplemental figure S3). In fact, type I interferons have been shown to induce CXCL10 and CXCL9 production in DCs and subsequently enhance their ability to stimulate CD8 +effector T cells.^59 60^ However, other IFN-modulated chemokines/cytokines couple be involved, potentially in a different manner in the tumor or LN.^61–64^ Thus, further exploration on how other chemokines/cytokines may be impacted by Alb-IFNβ should be considered.

Alb-IFNβ may serve as a protein based adjuvant that can be used to enhance protein based vaccines. It would be important to further test whether Alb-IFNβ can be used as an adjuvant to improve vaccine efficacy of other types of protein based vaccines or other forms of vaccine, such as DNA/RNA based, cell based, or vector based vaccines. This information would create the opportunity for wide application of Alb-IFNβ to enhance vaccine potency. For clinical translation, it will be important to further characterize the toxicity generated by Alb-IFNβ. The understanding of the ability of Alb-IFNβ to enhance vaccine potency as well as the toxicity associated with the coadministration of Alb-IFNβ will be critical for the assessment whether Alb-IFNβ will serve as a better adjuvant compared with other adjuvants. Such information will be critical for the development of vaccines against infections and cancers.

## Data Availability

Data are available on reasonable request. All data and materials are available from the corresponding author on written request.
