# Supplementary material for: Albumin and interferon-β fusion protein serves as an effective vaccine adjuvant to enhance antigen-specific CD8+ T cell-mediated antitumor immunity
Source: J Immunother Cancer. 2022 Apr 22;10(4):e004342. doi: 10.1136/jitc-2021-004342 (PMC9036441; doi:10.1136/jitc-2021-004342)
Supplement: Supplementary data [file jitc-2021-004342supp001.pdf]

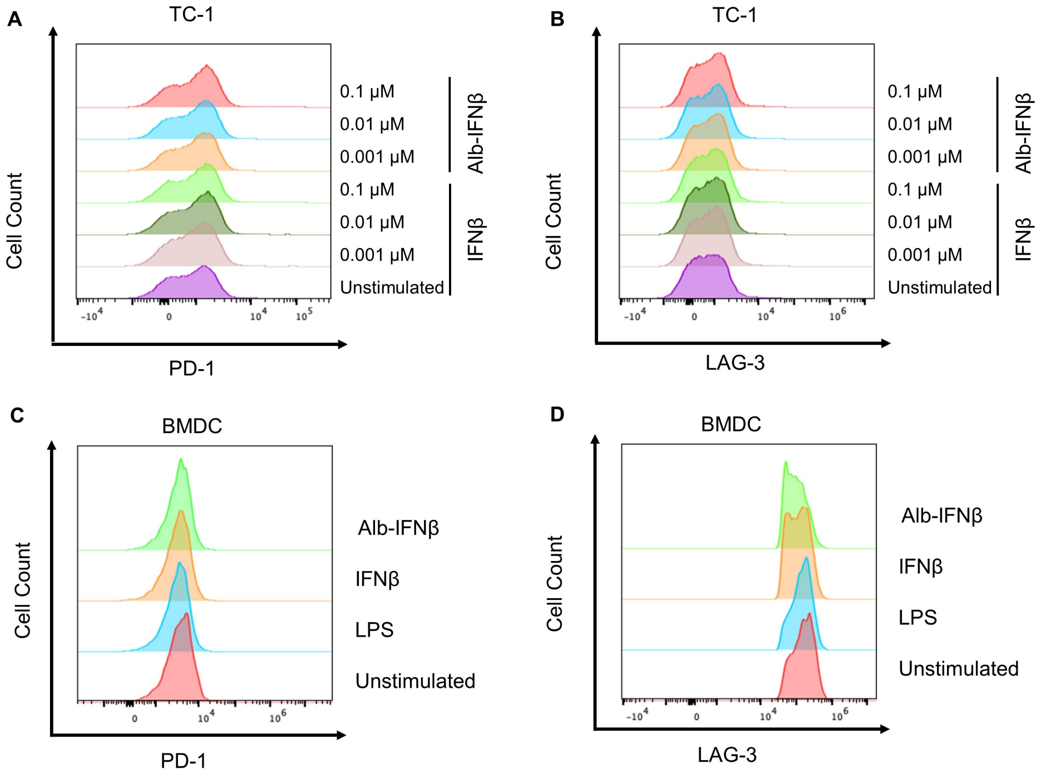

Supplemental Figure 1 Characterization of biological activity of Alb-IFNβ compared to IFNβ using TC-1 cells and BMDCs. TC-1 cells were treated with 0.1 μM, 0.01 μM, or 0.001 μM of either IFNβ or Alb-IFNβ for 24 hrs. The TC-1 cells were subsequently harvested and analyzed with flow cytometry for the expression of **(A)** PD-1 and **(B)** LAG-3 expression. BMDCs were treated with 0.1 μM of either IFNβ or Alb-IFNβ for 24 hrs. BMDCs treated with lipopolysaccharide (LPS) as positive control. Shown here are representative flow cytometry images of **(C)** PD-1 and **(D)** LAG-3 expression.
