# Supplementary material for: Albumin and interferon-β fusion protein serves as an effective vaccine adjuvant to enhance antigen-specific CD8+ T cell-mediated antitumor immunity
Source: J Immunother Cancer. 2022 Apr 22;10(4):e004342. doi: 10.1136/jitc-2021-004342 (PMC9036441; doi:10.1136/jitc-2021-004342)
Supplement: Supplementary data [file jitc-2021-004342supp002.pdf]

**Supplemental Figure 2**

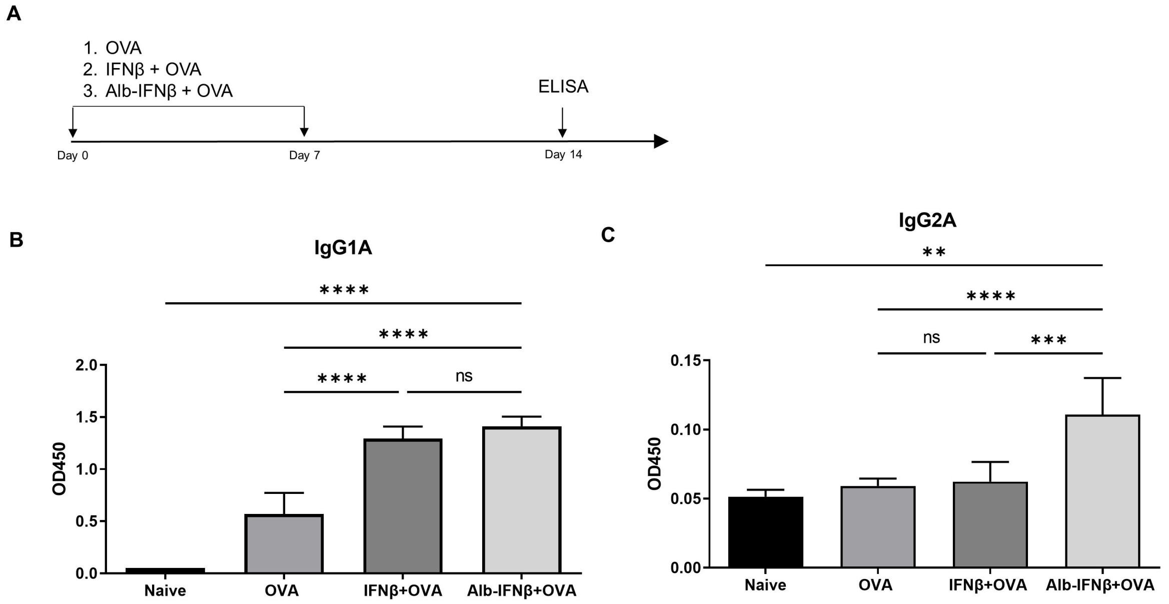


**Supplemental Figure 2. Co-administration of Alb-IFNβ and OVA leads to enhanced OVA specific IgG2A antibody response.** To detect mouse anti-OVA IgG1 or IgG2A antibodies in vaccinated mice, an ELISA assay was performed. Sera form vaccinated and untreated mice were collected on day 25. **(A)** Schematic illustration of the experiment (same as in **Figure 2**). Sera from naïve mice served as negative control. The sera were diluted 1000× fold. **(B)** ELISA results of IgG1 antibodies. **(C)** ELISA results of IgG2A antibodies. *p<0.05, **p<0.01, ***p<0.001, ****p< 0.0001, ns not signficiant.
