# Supplementary material for: Albumin and interferon-β fusion protein serves as an effective vaccine adjuvant to enhance antigen-specific CD8+ T cell-mediated antitumor immunity
Source: J Immunother Cancer. 2022 Apr 22;10(4):e004342. doi: 10.1136/jitc-2021-004342 (PMC9036441; doi:10.1136/jitc-2021-004342)
Supplement: Supplementary data [file jitc-2021-004342supp003.pdf]

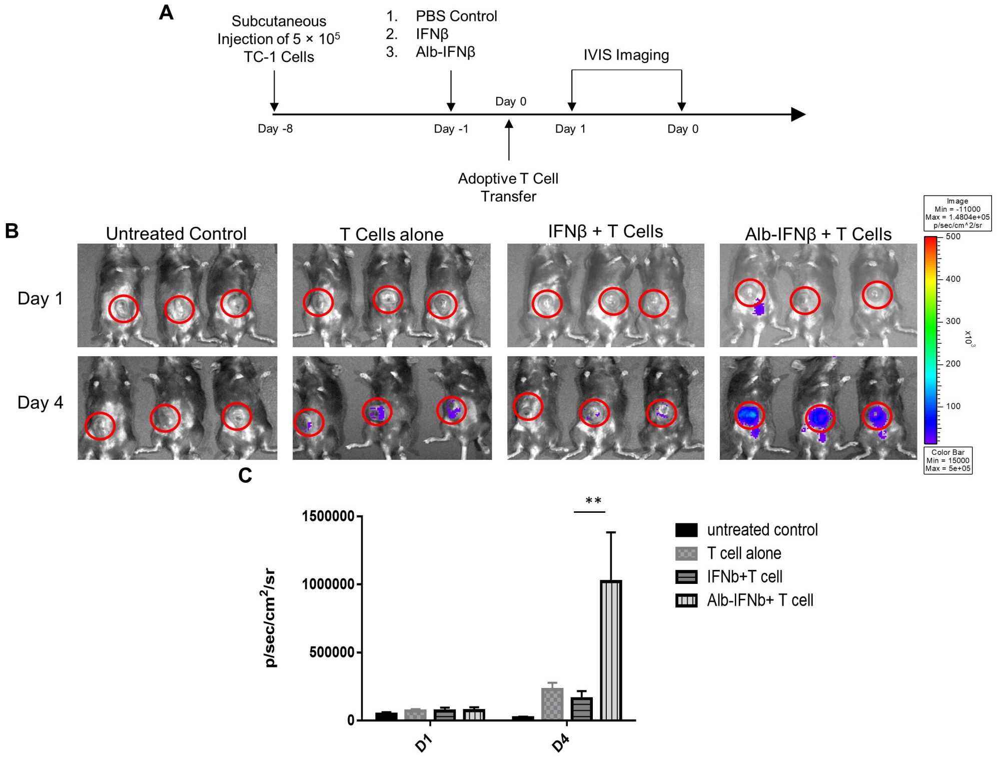

Supplemental Figure 3 Characterization of E7-specific CD8+ T cell infiltration in tumors following injection with Alb-IFNβ. (A) Schematic illustration of the experiment. Briefly, C57BL/6 mice (3 per group) were s.c. injected with 5 × 10^5 TC-1 cells 8 days prior to the adaptive transfer of luciferase-expressing E7-specific T cells. Tumor-bearing mice were i.v. injected with either PBS, IFNβ, or Alb-IFNβ one day before T cell transfer. On day 0, mice were i.v. adoptive transferred E7-specific T cells in indicated groups, and imaged by IVIS spectrum on days 1 and 4 after T cell transfer. **(B)** IVIS imaging of the mice. Tumor regions are indicated by red circles. **(C)** Bar graph displaying the mean fluorescence signals showing T cell infiltration into the tumor regions.
