# Supplementary material for: Albumin and interferon-β fusion protein serves as an effective vaccine adjuvant to enhance antigen-specific CD8+ T cell-mediated antitumor immunity
Source: J Immunother Cancer. 2022 Apr 22;10(4):e004342. doi: 10.1136/jitc-2021-004342 (PMC9036441; doi:10.1136/jitc-2021-004342)
Supplement: Supplementary data [file jitc-2021-004342supp004.pdf]

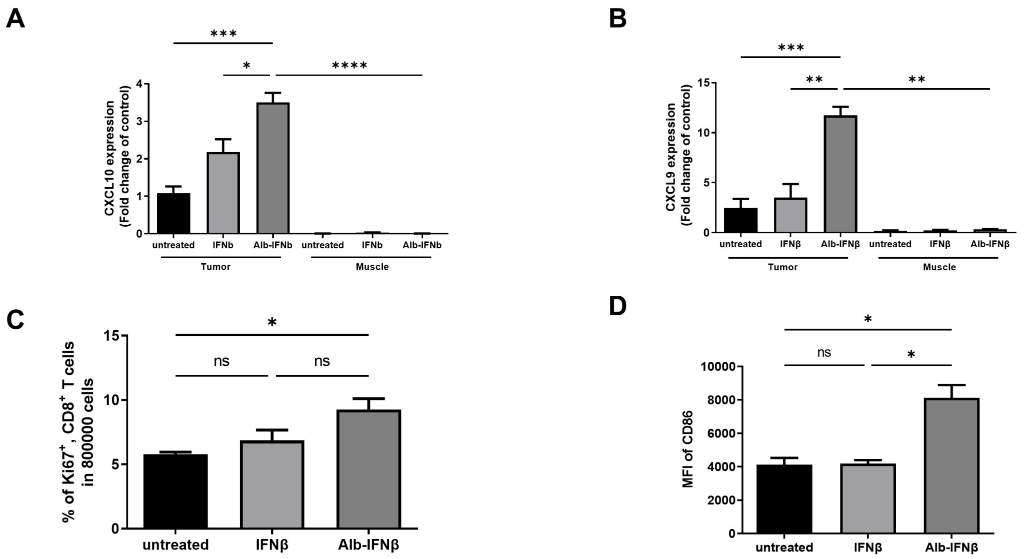

Supplemental Figure 4 Characterization of chemokine levels in tumors and immune cell activity in tumor draining lymph nodes following injection with Alb-IFNβ. Tumor-bearing mice (*n* = 3) were i.v. injected with either PBS, IFNβ, or Alb-IFNβ. Three days after injection, tumors and tdLNs were harvested for quantitative real-time PCR (qRT-PCR) or flow cytometric analyses. **(A)** qRT-PCR data of CXCL10 expression in tumors. **(B)** qRT-PCR data of CXCL9 expression in tumors. **(C-E)** Cells were isolated form tdLNs and stained for proliferative CD8+ T cells, or DC activation. **(C)** Bar graph summary of percentage of proliferative CD8+ T cells in tdLNs. **(D)** Bar graph summary of mean fluorescence intensity of CD86 in CD11c+ and I-A+/I-E+ DCs. *p<0.05, **p<0.01, ***p<0.001, ****p< 0.0001, ns not significant.
